# Protozoan parasites in *Culex pipiens* mosquitoes in Vienna

**DOI:** 10.1007/s00436-019-06219-8

**Published:** 2019-02-19

**Authors:** Ellen R. Schoener, Josef Harl, Tanja Himmel, Karin Fragner, Herbert Weissenböck, Hans-Peter Fuehrer

**Affiliations:** 1 Institute of Parasitology, Department of Pathobiology, University of Veterinary Medicine Vienna, Veterinaerplatz 1, 1210 Vienna, Austria; 2 Institute of Pathology and Forensic Veterinary Medicine, Department of Pathobiology, University of Veterinary Medicine Vienna, Veterinaerplatz 1, 1210 Vienna, Austria

**Keywords:** Avian *Plasmodium*, *Crithidia*, *Culex pipiens*, Mosquito vectors, *Trypanosoma*

## Abstract

Avian malaria (*Plasmodium* spp*.*) and kinetoplastid (*Trypanosoma* spp.) parasites are common vector-borne pathogens in birds worldwide; however, knowledge about vector competence of different mosquito species is currently lacking. For a pilot project examining vector competence of mosquitoes of the *Culex pipiens* complex and *Culex torrentium* for protozoan parasites in the city of Vienna, 316 individual mosquitoes were sampled in the months June–August 2017 around the campus of the Veterinary University of Vienna. Since vector competence for avian *Plasmodium* can only be ascertained by finding infectious sporozoites in mosquito salivary glands, special emphasis was on examining these, or at least insect thoraxes, which contain the salivary glands. After species identification, the mosquitoes were processed in three different ways to determine the best method of visually detecting protozoan parasites in salivary glands: (1) microscopic examination of individual, fixed and Giemsa-stained salivary glands, (2) microscopic examination of stained sections of individually fixed and embedded mosquito thoraxes and (3) stained sections of individual whole insects. Material from all three groups was also subjected to PCR to detect avian haemosporidian and trypanosomatid parasite DNA. PCR was performed on all 316 collected mosquitoes, with 37 pools (*n* = 2–10) of 263 individuals and 53 single individuals in all together 90 PCR reactions. Avian *Plasmodium* was found in 18 (20%) and trypanosomatid parasites were found in 10 (11.1%) of the examined samples and pools yielded a higher proportion of positives than did individual samples. Six different species of protozoan parasites were identified, namely *Plasmodium vaughani* SYAT05 which was the most common, *P. elongatum* GRW6, *P. relictum* SGS1, *Trypanosoma avium*, *T. culicavium* and *Crithidia dedva*. Seventy-seven mosquito salivary glands were dissected and stained with Giemsa solution. Of these, one (1.3%) featured sporozoites and one (1.3%) trypanosomatid parasites. While the trypanosomes were identified as *T. avium*, the avian *Plasmodium* species were present in a mixed infection with *P. vaughani* SYAT05 as the dominant species. In conclusion, mosquitoes of the *Culex pipiens* complex are very likely vectors of different avian *Plasmodium* and *Trypanosoma* species and PCR was the most successful and reliable method for parasite detection in mosquito samples, delivering higher rates and more accurate results. The visual detection of parasite stages in the salivary glands was more difficult and only a few specimens were detected using Giemsa stain and chromogenic in situ hybridization. For further studies on vector competence of different protozoan parasites in mosquitoes, the use of PCR-based methods would be preferable.

## Introduction

Avian malaria (*Plasmodium* spp*.*) and kinetoplastid (*Trypanosoma* spp.) parasites are common vector-borne pathogens in birds worldwide. While avian trypanosomes are mainly considered harmless for their hosts (Šlapeta et al. 2016), avian malaria parasites have the ability to cause disease and mortality, and infections can therefore become a serious issue for bird conservation (Atkinson and Van Riper III 1991). In Hawaii, *Plasmodium relictum* (lineage GRW4) caused mortality and extinction of many endemic bird species after the arrival of Europeans who inadvertently introduced the necessary vectors. Insect vectors, namely mosquitoes of the dipteran family Culicidae, are compulsory for *Plasmodium* parasites to complete their life cycle and for the transmission of some avian trypanosomes (Šlapeta et al. 2016) and therefore should not be overlooked when studying diversity, infectivity and epidemiology of these organisms*.* Despite this, mosquitoes as vectors are only just now in the past decade been studied in detail and baseline data is now emerging.

For most species of *Plasmodium*, the insect hosts are known to be mosquitoes of the genera *Culex*, *Aedes* and *Anopheles* (Valkiūnas 2005). The vector competence for transmitting avian malaria varies between mosquito species, and each *Plasmodium* species may use a number of different mosquito species as vectors (Kimura et al. 2010). To date, a list of specific vectors for avian *Plasmodium* spp. (Glaizot et al. 2012; Valkiūnas 2005) and trypanosomes has not yet been determined. So far, the genus *Culex* seems to comprise the most successful avian malaria vectors worldwide and also appears to be involved in the transmission of some avian trypanosomes (Šlapeta et al. 2016; Votýpka et al. 2012); various studies showed that *Culex* mosquitoes contained the biggest diversity of different *Plasmodium* strains (Glaizot et al. 2012; Kimura et al. 2010). In Europe, experimental evidence points to *Cx*. *pipiens* s.l. (Kazlauskienė et al. 2013) and *Cx. pipiens* f. *molestus* (Ziegyte et al. 2014) as very likely competent vectors for the *P. relictum* lineages pSGS1 and pGRW11 and GRW4 (Ionica et al. 2017; Valkiūnas et al. 2015). Knowledge about vector competence is currently lacking for other European mosquito species (Santiago-Alarcon et al. 2012).

In previous years (2013–2015), our work group collected 45,749 mosquitoes in Eastern Austria (Burgenland, Lower Austria and Vienna). These mosquitoes were examined for avian *Plasmodium* (Schoener et al. 2017) and trypanosomes (Schoener et al. 2018). For avian *Plasmodium*, 169 (6.43%) of 2628 pools (of up to 50 individuals) were positive, with the majority of positives in mosquitoes of the morphologically indistinguishable *Culex pipiens* complex and *Cx. torrentium*, which had a prevalence of 15.4%. Six different avian *Plasmodium* lineages were found, the most common were *P. vaughani* SYAT05, *P. matutinum* LINN1 and *P. relictum* SGS1. For avian trypanosomes, 96 (15.6%) of the 616 examined pools of the *Culex pipiens* complex were positive, with most positives for *Trypanosoma culicavium*, a parasite which was first described in 2012 (Votýpka et al. 2012).

The avian *Plasmodiu*m stages can be found in the midgut and salivary gland of the vector (Valkiūnas 2005). Sexual reproduction of these parasites takes place in the midgut of the mosquito and after its completion, sporozoites are formed which migrate from the insect’s haemolymph to the salivary glands, from where they are transmitted to the vertebrate host in competent vectors (Valkiūnas 2005). To find an infection in the vector, the insect has to be carefully dissected and examined by histological (to visualize the parasites) or molecular techniques (to detect their DNA). PCR-based methods have improved the detectability of haemosporidian infections in birds (Krizanaskiene et al. 2006). Owing to their large number of molecular targets, DNA-based diagnostics have the ability to detect parasites at densities too low for detection by conventional microscopy (Freed and Cann 2003). The most commonly used standard PCR for avian *Plasmodium* diagnostics was developed by Hellgren et al. (2004) and amplifies a part of the parasite’s cytochome b (CytB) gene. Data on most avian haemosporidian CytB lineages with codes assigned to individual haplotypes are listed in the global avian malaria database MalAvi (Bensch et al. 2009). There is an important problem with the standard nested PCR, especially when looking at pooled mosquito samples. In pooled samples, there are often more than one *Plasmodium* species present, but the conventional PCR assay underestimates mixed infections, because the DNA of some parasites is amplified better than that of others (Valkiūnas et al. 2006). In 2011, a chromogenic in situ hybridization (ISH) procedure was established to detect avian *Plasmodium* parasites in paraffin wax-embedded tissues of birds (Dinhopl et al. 2011). This method uses a probe targeting the 18S rRNA and reliably allows for the detection of *Plasmodium* blood and tissue cell stages in deceased penguins and passerine birds (Dinhopl et al. 2011, 2015). So far, only a general probe is available which cannot differentiate between different *Plasmodium* species or lineages. The method also has not been tested in insect vectors, although it is very sensitive and can detect a variety of different developmental stages and should therefore also aid with the detection of parasites in insect vector tissue.

In order to prove vector competence in mosquitoes, two strategies are available, either experimental infections between vertebrate and invertebrate host with controlled settings in a laboratory or the detection of infectious stages in the salivary glands of the insects (either field- caught or laboratory raised). Experimental infections require a large logistical and financial effort, including high security level laboratories and animal ethics for experimental animals (birds in this case), but the detection of infectious parasite stages in salivary glands mainly requires time and patience. After their development in the vector, not all avian *Plasmodium sporozoites* enter the salivary glands and therefore become infectious. Studies on human *Plasmodium* parasites have shown that in *P. vivax*, only around 20% invade the salivary glands of the vector (Rosenberg and Rungsiwongse 1991), while in *P. falciparum*, 11.3% fail to penetrate the salivary glands and instead remain in the haemolymph (Vaughan et al. 1992). It is therefore important to dissect the salivary glands instead of examining whole insect samples.

To determine the best method for visually detecting protozoan parasite stages in salivary glands of mosquito vectors, dissection and histological analysis of fresh *Culex pipiens* mosquito specimens were performed.

## Material and methods

### Mosquito sampling

Mosquitoes were sampled from June to August 2017 at the campus of the University of Veterinary Medicine Vienna (48.252302° N, 16.428255° E) as well as the neighbouring Theresa Tauscher Park (48.260377° N, 16.429378° E), due to the close proximity, both sites were combined. Each month, mosquitoes were collected once at a permanent sampling site at the university campus for a 24-h time period using a Biogents Sentinel Trap (Regensburg, Germany) equipped with bottled carbon dioxide (Air Liquide, Schwechat, Austria) as attractant. Gravid female mosquitoes were collected several times a week (weather dependent) using six Biogents gravid traps. These traps were set up containing fermented hay water in the afternoon, and mosquitoes were collected the following morning. Mosquitoes were killed using over the counter insecticide (Ameisen-Power-Spray, Vandal, Vienna, Austria) upon entering the gravid trap. In addition, blood-fed and questing female mosquitoes were collected on the campus of the University of Veterinary Medicine Vienna using insect aspirators. The collected mosquitoes were processed in the lab within 1 day after collection.

### Morphological identification and DNA extraction

Morphological identification of *Culex pipiens* mosquitoes was performed using the identification key of Becker et al. (2010). To determine the best method for visually detecting avian malaria sporozoites, the mosquitoes were processed in three different ways:

1. Mosquito salivary glands (*n* = 77) were extracted on individual glass slides under a dissection microscope as described by Valkiūnas (2005), and the ruptured salivary glands were air-dried, fixed for 1 min in absolute methanol and stained with Giemsa solution.
2. Mosquito thoraxes (*n* = 196), where the salivary glands are located, were separated and fixed individually in 4% formalin solution overnight and then embedded in paraffin wax for further histological processing and staining.

For both of the above, the remaining insect parts (remaining thorax tissues and abdomens) were pooled by date in pools of one to ten individuals and processed for DNA extraction and PCR.

3. Individual mosquitoes (*n* = 43) were fixed whole in 4% formalin solution overnight and then embedded in paraffin wax for further histological processing and staining. From these paraffin blocks, slices of not more than 25 mg, containing whole insect tissue, were used for DNA extraction according to the protocol for pre-treatment for paraffin- embedded tissue of the Qiagen DNeasy blood and tissue kit (Qiagen, Hilden, Germany).

DNA was extracted from all samples using the Qiagen DNeasy Blood and Tissue kit (Qiagen, Hilden, Germany). Each sample was placed in 180 μl buffer ATL and 20 μl proteinase K with two ceramic beads (Precellys Ceramic Beads, Peqlab Biotechnologie GmbH, Erlangen, Germany) and homogenized in a Qiagen TissueLyser II. The homogenized material was loaded onto a QIAshredder. To filter the samples, the filled QIAshredders were centrifuged for 2 min at 13,000 rpm (solid components of the samples remained on the column). The samples were then incubated at 56 °C overnight and processed according to the manufacturer’s protocol.

### PCR, sequencing and sequence analysis

For detecting the presence of *Plasmodium* spp., each DNA sample was then subjected to nested PCR (Hellgren et al. 2004). The used primers target a 480-bp fragment of the mitochondrial CytB gene. For amplifying trypanosomatid parasite DNA, each DNA sample was subjected to nested PCR (Seward et al. 2017). The used primers target a ~ 2000-bp fragment of the nuclear ribosomal small subunit (SSU) gene. PCR products were separated by electrophoresis in 2% agarose gels stained with Midori Green Advance DNA stain (Nippon Genetics Europe, Germany). Finally, purified PCR products were commercially sequenced at LGC Genomics GmbH, Germany. Obtained sequences were viewed and aligned using the programme Genious version 10.0.6 (http://www.geneious.com) (Kearse et al. 2012). Then, the sequences were compared for similarity to sequences available on the MalAvi (http://mbio-serv2.mbioekol.lu.se/Malavi/) and the GenBank® (http://www.ncbi.nlm.nih.gov/BLAST) databases. Amplifying parasite DNA from mosquito pools and from body regions apart from isolated salivary glands is no proof for vector competence, since infectious sporozoites are only found in the salivary glands, and findings in the thorax, for example, are only indicative for a vector role.

Three legs of each mosquito were taken and processed individually to identify the species/biotypes of *Cx. pipiens* s.l./*Cx. torrentium* genetically as described in a previous study (Zittra et al. 2016).

Blood-fed mosquito individuals were subjected to a PCR described by Kent (2009), Njabo et al. (2011) and Njabo et al. (2009) to identify the vertebrate host, using the general vertebrate primers L14724 (5′-CGAAGCTTGATATGAAAAACCATCGTTG-3′) (Irwin et al. 1991) and H15149 (5′-AAACTGCAGCCCCTCAGAATGATATTTGTCCTCA-3′) (Kocher et al. 1989), as well as the avian primers Avian b F (5′-CCCTCAGAATGATATTTGTCCTCA-3′) and Avian b R (5′-CCTCAGAAKGATATYTGNCCTCAKGG-3′) (Kent 2009; Molaei et al. 2006).

Paraffin-embedded material of PCR positives was cut in 3 μm sections, mounted on slides and stained with haematoxylin and eosin (HE), and avian *Plasmodium* positives were in addition stained with a previously established chromogenic in situ hybridization (ISH) technique (Dinhopl et al. 2011).

All stained slides were screened using a light microscope and up to × 1000 magnification to visualize avian *Plasmodium sporozoites*.

## Results

During the months June to August 2018, 316 mosquitoes of the *Culex pipiens* complex and *Cx. torrentium* were collected. The majority of individuals (*n* = 249, 78.78%) was caught using a BG sentinel trap, followed by gravid trap (*n* = 39, 12.34%) and insect aspirator (*n* = 28, 8.86%) (Table 1). Of the 132 genetically identified mosquitoes, 126 (95.45%) were *Cx. pipiens* f*. pipiens*, three (2.27%) were *Cx. pipiens* f. *pipiens*/*molestus* hybrids, two were *Cx. pipiens* f*. molestus* (1.52%) and one (0.76%) was *Cx. torrentium* (Table 2).

**Table 1:** Results of different sampling methods for the *Culex pipiens* complex in summer 2018 (total individuals *n* = 316)

	Insect aspirators	Gravid trap	BG sentinel trap
June	0	13	43
July	15	13	94
August	13	13	112
Total	28	39	249

**Table 2:** Forms of the *Culex pipiens* complex collected in the months June–August 2018 (total individuals *n* = 132)

	*Culex pipiens* f. *pipiens*	*Culex pipiens* f. *molestus*	*Culex pipiens/molestus* hybrid	*Culex torrentium*
June	29	0	2	1
July	46	2	1	0
August	50	0	0	0
Total	126	2	3	1

To identify protozoan parasites, PCRs for both avian haemosporidian and trypanosomatid parasites were performed on all 316 collected mosquitoes (37 pools of 263 individuals and 53 single individuals; Table 3). Avian *Plasmodium* was found in 18 (20%) and trypanosomatid parasites were found in 10 (11.11%) of the examined samples and pools yielded a higher proportion of positives than did individual samples (Table 4). Six different species of protozoan parasites were identified, namely *P. vaughani* SYAT05 which was also the most common, *P. elongatum* GRW6, *P. relictum* SGS1, *T. avium s.l.*, *T. culicavium* and *Crithidia dedva* (Table 5). The BLAST search revealed that the trypanosomatid parasites *T. avium* s.l. and *T. culicavium* featured haplotypes previously found in *Cx. pipiens* mosquitoes (Votýpka et al. 2012; Zidkova et al. 2012).

**Table 3:** PCR positives for protozoan parasites in the months June–August 2018

	Avian *Plasmodium*	Trypanosomatid parasites
June: *n* positives/tested (%)	5/11 (45.45%)	4/11 (36.36%)
July: *n* positives/tested (%)	6/35 (17.14%)	3/35 (8.57%)
August: *n* positives/tested (%)	7/44 (15.9%)	3/44 (6.82%)
Total: *n* positives/tested (%)	18/90 (20%)	10/90 (11.11%)

**Table 4:** Detection of protozoan parasites using different methods (total individuals *n* = 316)

Parasites	PCR (*n* = 90; 37 pools of 263 individuals, 53 single individuals)		Giemsa (*n* = 77)		ISH of PCR positives (*n* = 35)
	Avian *Plasmodium*	Trypanosomatid parasites	Avian *Plasmodium sporozoites*	Trypanosomatid parasites	Avian *Plasmodium*
*n* positives/tested individuals (%)	7/53 (13.21%)	3/53 (5.66%)	1/77 (1.3%)	1/77 (1.3%)	2/35 (5.7%)
*n* positives/tested pools	11/37 (29.73%)	7/37 (18.92%)	N/A	N/A	N/A
Positives/total	18/90 (20%)	10/90 (11.11%)	1/77 (1.3%)	1/77 (1.3%)	2/35 (5.7%)

**Table 5:** Protozoan parasites found in *Culex pipiens* complex and *Cx. torrentium* in summer 2018 during the months June–August 2018

	Avian *Plasmodium*						Trypanosomatid parasites				
	Total	Mix (*P. vaughani* SYAT05 dominant)	*P. vaughani* SYAT05	*P. elongatum* GRW6	*P. relictum* SGS1	Mix (unidentified *Plasmodium*)	Total	*T. avium s.l.*	*T. culicavium*	*Crithidia dedva*	Unidentified
June	5	4	1				4	1	2		1
July	6		2		2	2	3	2			1
August	7	2	4	1			3	1	1	1	
Total	18	6	7	1	2	2	10	4	3	1	2

A total of 20 blood-fed individuals were collected, which all were *Cx. pipiens* f. *pipiens*. Of these, 12 contained vertebrate DNA, with seven yielding usable sequences for further analysis of which six showed *Homo sapiens* and one *Turdus merula* (European blackbird) as vertebrate hosts. The mosquito with *T. merula* blood was also positive for *P. vaughani* SYAT05 (Table 5).

**Fig. 1:**
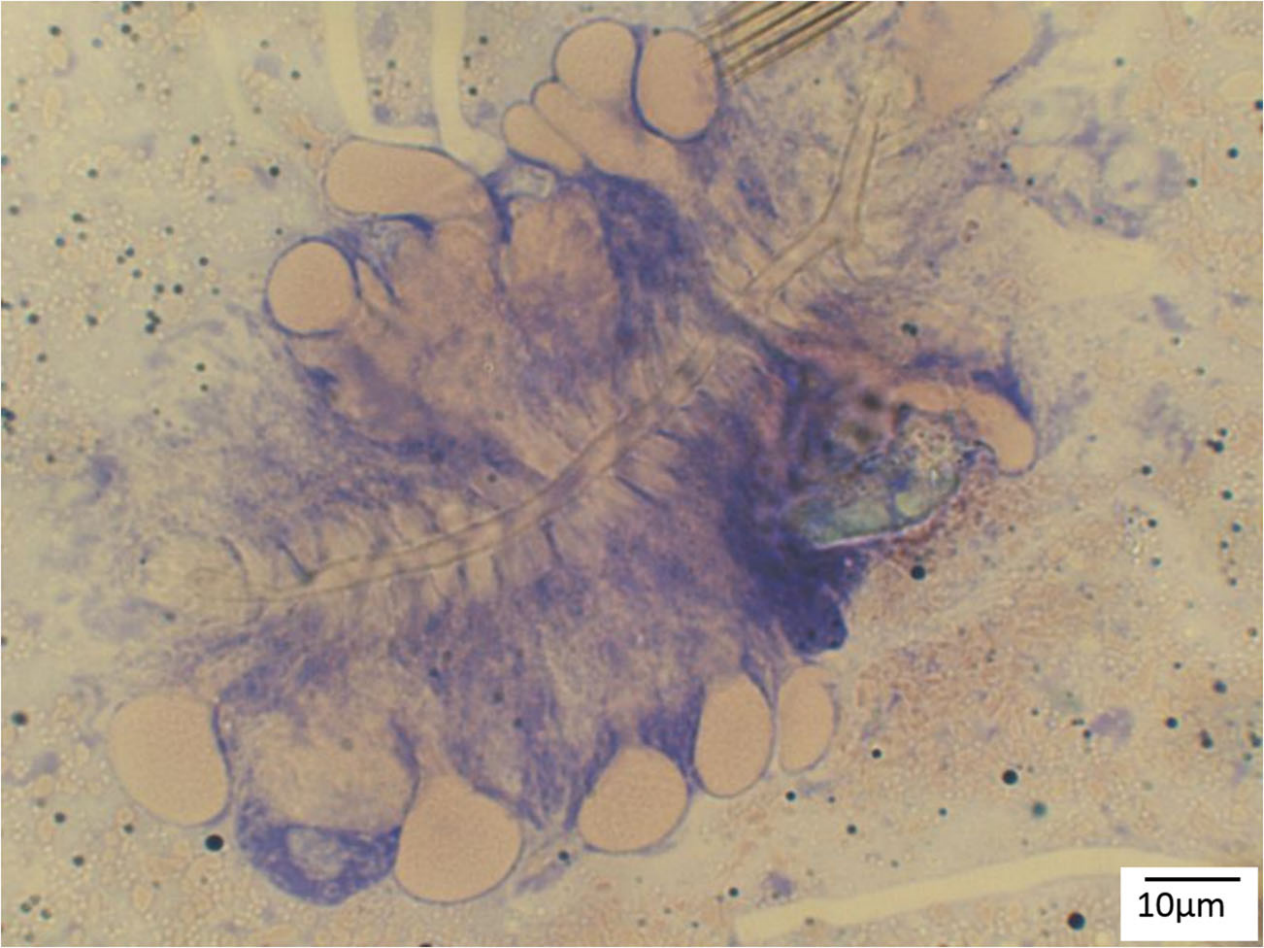
*Culex pipiens* s.l. salivary gland stained with Giemsa (× 400 magnification)

**Fig. 2:**
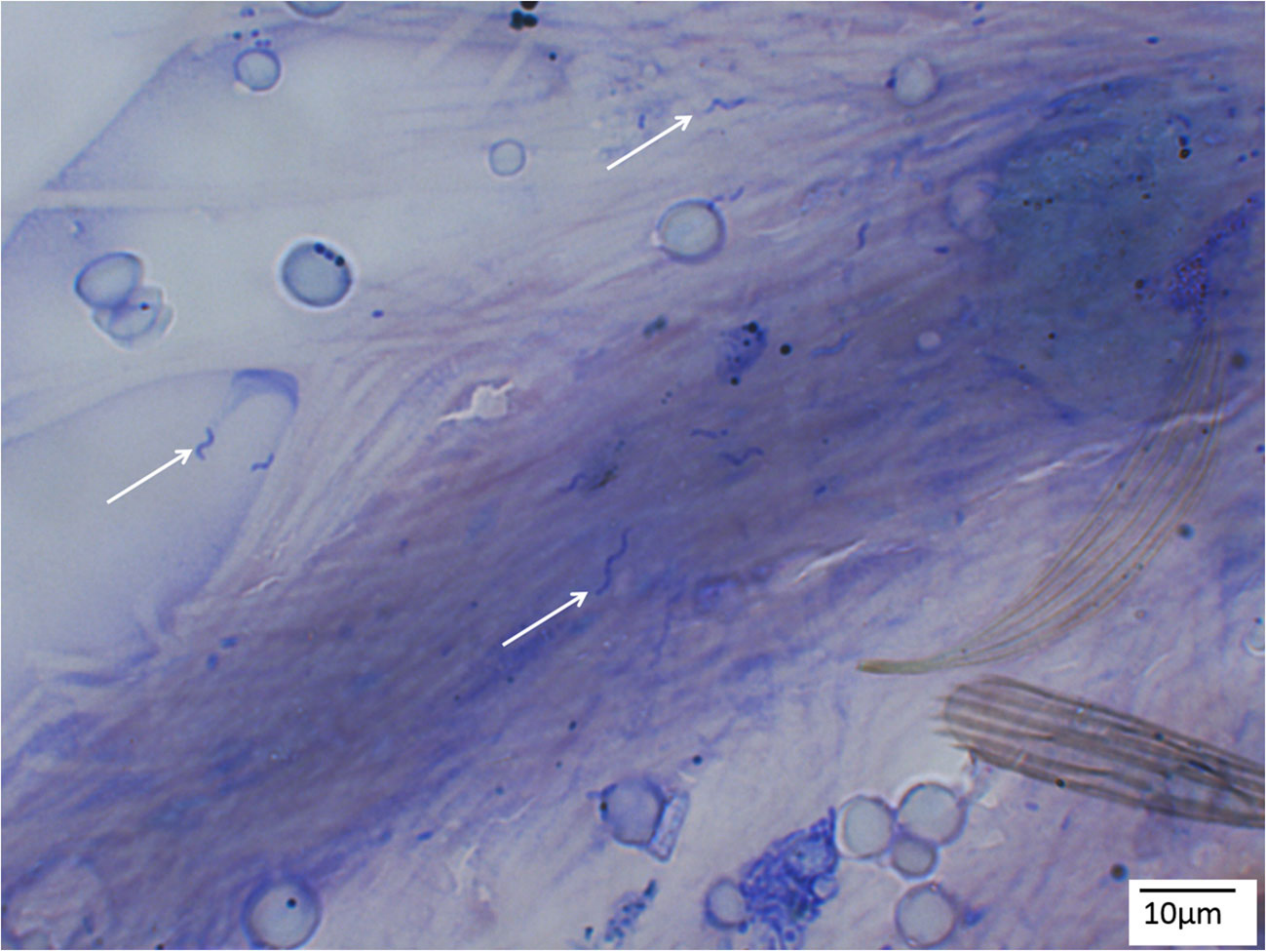
Avian *Plasmodium* sporozoites (arrows) from *Culex pipiens* s.l. under × 1000 magnification (Giemsa stain)

**Fig. 3:**
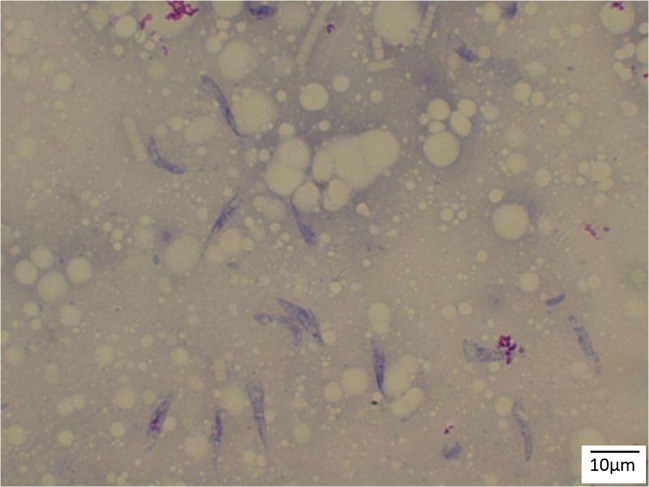
*Trypanosoma avium* from *Cx.pipiens* f. *pipiens* salivary glands (Giemsa stain, × 1000 magnification)

To detect avian *Plasmodium sporozoites*, 77 mosquito salivary glands were dissected and stained with Giemsa stain (Fig. 1). Of these, one (1.3%) presented with sporozoites (Fig. 2) and one *Cx. pipiens* f. *pipiens* (1.3%) showed trypanosomatid parasites (Fig. 3). While the trypanosomes were identified as *T. avium*, the avian *Plasmodium* species were present in a mixed species infection, visible in the electropherograms, and although *P. vaughani* SYAT05 peaks were dominant, the other species could not be identified.

**Fig. 4:**
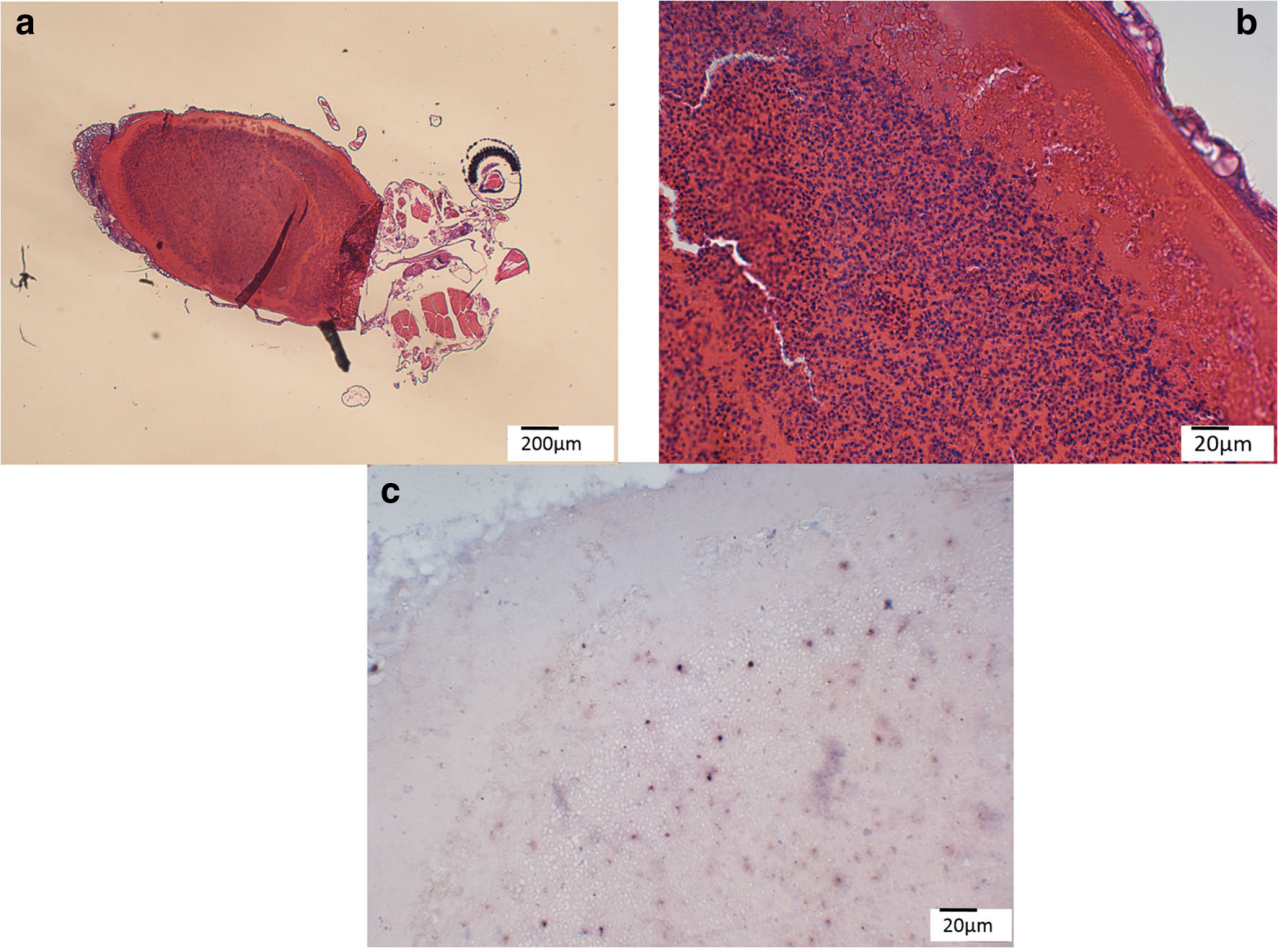
Detection of avian *Plasmodium* in the blood-filled abdomen of a *Cx. pipiens* f*. pipiens* mosquito using ISH. **a** The whole individual under × 40 magnification. **b** Nucleated erythrocytes are visible in the partially digested blood meal. **c** The distinct purple to black signal of avian *Plasmodium* blood stages using ISH

ISH for avian *Plasmodium* was performed on all embedded (whole and thorax) PCR positives (*n* = 35) and of the 35 prepared slides, two (5.7%) presented with the distinct purple to black signal in the blood filled abdomen (Fig. 4). Both mosquito individuals (*Cx. pipiens* f. *pipiens*) had nucleated blood cells (indicative for avian blood) in the abdomen and were sequence positive for *P. relictum* SGS1, but only human DNA was amplified during blood meal identification.

## Discussion

Mosquito collection and the proportion of *Culex pipiens* complex and *Cx. torrentium* found in this study mimicked very closely recent findings in the city of Vienna (Schoener et al. 2017, 2018). In our preceding, much larger, study on parasites of mosquitoes in Vienna, a higher diversity of mosquitoes was recorded including several thousand individuals of the *Cx. pipiens* complex. Previously, the prevalence of avian *Plasmodium* in pools of *Cx. pipiens* s.l. in Vienna ranged from 42.57% (2014) to 53.16% (2015) featuring five different species of avian *Plasmodium* as well as one species of *Leucozytozoon*, although the most prevalent species was *P. vaughani* SYAT05 followed by *P. relictum* SGS1 (Schoener et al. 2017), which was also seen in the present study. For trypanosomatid parasites, the prevalence in previous years ranged from 20.79% (2014) and 32.11% (2015) in *Cx. pipiens* mosquitoes, with four different species of trypanosomatids detected and *T. culicavium* as the most common (Schoener et al. 2018). In addition to the previously found species, another monoxenous trypanosomatid parasite, *Crithidia dedva*, was found in one pool in August 2017. The difference in parasite prevalence between previous years and 2017 is most likely due to the examined material. While in the years 2014 and 2015, large pools of up to 50 individuals were examined; the present study examined individuals and smaller pools of up to 10 individuals only, with the probability of a positive individual in a smaller pool being less likely.

Only a few of our collected mosquitoes were blood fed. Blood meal identification of the vertebrate host is possible but depends on the state of digestion (Kent 2009). The commonly used vertebrate primers L14724 (Irwin et al. 1991) and H15149 (Kocher et al. 1989) for mosquito blood meal identification (Njabo et al. 2009) amplify vertebrate DNA reliably, but appear to preferentially amplify mammal over bird DNA (Molaei et al. 2006). This was also the case in our study where most of our blood-fed mosquitoes presented with human blood, although nucleated erythrocytes were visible in the mosquito abdomen under the microscope. We repeated the PCR using the avian primers Avian b and Avian b R (Kent 2009; Molaei et al. 2006), but were only in one case able to amplify blackbird DNA. For future studies, care has to be taken when performing blood meal identification using just PCR, since our microscopic evidence showing nucleated erythrocytes points to at least two blood meals taken by the mosquito, one from a human (PCR result) and one from a bird. The mosquito in our study with *T. merula* blood was also positive for *P. vaughani* SYAT05, having most likely just taken a blood meal from an infected blackbird.

While PCR-based methods during this study delivered reliable and detailed results about protozoan parasite DNA present in the examined *Cx. pipiens* mosquitoes, the visual detection of parasite stages was more difficult and only few were detected using Giemsa stain and ISH. The preparation of histological slides of mosquito material proved to be difficult, because mosquito material embedded in paraffin showed to be brittle and many mounted mosquito sections did not show mosquito internal organs because these had broken away. The detection of *Plasmodium* sporozoites using the ISH probes developed previously (Dinhopl et al. 2011) could not be achieved either, although blood stages in blood-fed mosquitoes were found. The reason for this is unknown, but could possibly be due to differences in ribosomal structure and RNA sequences in sporozoites which have been shown in certain human malaria parasites (Gunderson et al. 1987; Rogers et al. 1996). The use of ISH probes for the detection of avian *Plasmodium* on mosquito material therefore needs to be optimized.

In addition, DNA extracted from the paraffin blocks of embedded whole individual mosquitoes showed comparatively less positive results, most likely due to the fixation with formalin overnight. It is likely that these samples contained less DNA of lesser quality than the DNA samples extracted with the standard protocol.

This was a pilot study with a limited scope concerning available funding, logistics and personnel. Examining individual mosquitoes and even individual salivary glands by molecular methods, in addition to the work presented here (which was mainly examining pooled samples), would have provided valuable further information. Further larger scale studies are needed to confirm vector competence in different forma of the *Culex pipiens* complex. The results presented here are therefore limited to showcasing the difficulties of the visual detection of infectious parasite stages in mosquitoes and in providing further evidence of vector roles of these mosquitoes.

## Conclusion

In this pilot study, different methods for the detection of protozoan parasites in *Culex pipiens* mosquitoes were trialed to examine vector competence. Mosquitoes of the *Culex pipiens* complex are very likely vectors of different avian *Plasmodium* and *Trypanosoma* species, and PCR was the most successful and reliable method, delivering the highest detection rate and precise results. The visual detection of parasite stages was more difficult and only a couple were detected using Giemsa stain and chromogenic ISH. For further studies on the vector competence of different protozoan parasites in mosquitoes, the use of PCR based methods would be preferable. Ideally, PCR should be performed on individual salivary glands while others could be stained with Giemsa. The ISH technique needs to be optimized for *Plasmodium* stages present in mosquitoes and would remain the “icing on the cake”.
